# Colistin potentiation in multidrug-resistant *Acinetobacter baumannii* by a non-cytotoxic guanidine derivative of silver

**DOI:** 10.3389/fmicb.2022.1006604

**Published:** 2023-01-04

**Authors:** Deepak Kumar, Chaitali Singhal, Manisha Yadav, Pooja Joshi, Priyanka Patra, Subhash Tanwar, Amitava Das, Sumit Kumar Pramanik, Susmita Chaudhuri

**Affiliations:** ^1^Translational Health Science and Technology Institute (THSTI), Faridabad, India; ^2^CSIR-Central Salt and Marine Chemicals Research Institute, Bhavnagar, India; ^3^Indian Institute of Science Education and Research Kolkata, Mohanpur, India

**Keywords:** colistin resistance, *Acinetobacter baumannii*, antibiotic, potentiators, nano-formulation, silver, guanidinium

## Abstract

A novel nano-formulation (NF) that sensitizes *Acinetobacter baumannii* (AB) to otherwise ineffective colistin is described in the present study. Infections due to multidrug resistant (MDR) AB represent a major therapeutic challenge, especially in situations of pre-existing colistin resistance (colR). Subsequently, boosting the effectiveness of colistin would be a better alternative tactic to treat AB infections rather than discovering a new class of antibiotics. We have previously demonstrated an NF comprising self-assembled guanidinium and ionic silver nanoparticles [AD-L@Ag(0)] to have anti-biofilm and bactericidal activity. We report NF AD-L@Ag(0) for the very first time for the potentiation of colistin in Gram-negative colistin-resistant bacteria. Our results implied that a combination of clinically relevant concentrations of colistin and AD-L@Ag(0) significantly decreased colistin-resistant AB bacterial growth and viability, which otherwise was elevated in the presence of only colistin. In this study, we have described various combinations of minimum inhibitory concentration (MIC) of colistin (MICcol, ^1/2^ MICcol, and ^1/4^ MICcol) and that of AD-L@Ag(0) [MICAD-L@Ag(0), ^1/2^ MICAD-L@Ag(0), and ^1/4^ MICAD-L@Ag(0)] and tested them against MDR AB culture. The results (in broth as well as in solid media) signified that AD-L@Ag(0) was able to potentiate the anti-microbial activity of colistin at sub-MIC concentrations. Furthermore, the viability and metabolic activity of bacterial cells were also measured by CTC fluorescence assay and ATP bioluminescence assay. The results of these assays were in perfect concordance with the scores of cultures (colony forming unit and culture turbidity). In addition, quantitative real-time PCR (qRT-PCR) was performed to unveil the expression of selected genes, DNAgyrA, DNAgyrB, and dac. These genes introduce negative supercoiling in the DNA, and hence are important for basic cellular processes. These genes, due to mutation, modified the Lipid A of bacteria, further resisting the uptake of colistin. Therefore, the expression of these genes was upregulated when AB was treated with only colistin, substantiating that AB is resistant to colistin, whereas the combinations of MICcol + MICAD-L@Ag(0) downregulated the expression of these genes, implying that the developed formulation can potentiate the efficiency of colistin. In conclusion, AD-L@Ag(0) can potentiate the proficiency of colistin, further enhancing colistin-mediated death of AB by putatively disrupting the outer membrane (OM) and facilitating bacterial death.

## Introduction

*Acinetobacter baumannii* (AB) is an opportunistic Gram-negative bacillus responsible for causing drug-resistant nosocomial infections, including surgical wound infections, pneumonia, meningitis, and urinary tract infections (Moffatt et al., 2010). This notorious bacterium is resistant to many disinfectants and could form biofilms on abiotic surfaces. This ability thereby allows the bacteria to subvert innate immune defenses (Papathanakos et al., 2020). It has been ranked as the most critical target by the World Health Organization (WHO) (Tacconelli et al., 2018); the Food and Drug Administration (FDA) (Moffatt et al., 2010), and the Centers for Disease Control (CDC) (Centers for Disease Control and Prevention [CDC], 2019) for the development of anti-microbial agents (Geisinger et al., 2020). This is because, recently, isolated strains of AB are resistant to almost all antibiotics, such as, β-lactams, fluoroquinolones, tetracyclines, or aminoglycosides (Peleg et al., 2008), and this multidrug resistance has been on a continuous rise since last two decades (Munoz-Price et al., 2013; Qureshi et al., 2015). Carbapenems were thought to be the most appropriate agents to treat these multidrug resistant (MDR) AB strains (Dinc et al., 2013; Qureshi et al., 2015). However, resistance to carbapenems has been reported in AB worldwide, primarily due to the spread of international clones (Kim et al., 2014). Currently, this MDR/carbapenem-resistant bacteria is only treated with colistin methanesulfonate (CMS), which is an inactive prodrug that is converted to active drug colistin sulfate in blood (Velkov et al., 2013). Colistin (akin to polymyxin B) is a positively charged polypeptide antibiotic that targets multiple Gram-negative bacteria (GNB) (Evans et al., 1999; Li et al., 2006; Moffatt et al., 2010). These cationic antimicrobials target the negatively charged lipid A, which is the endotoxic component of lipopolysaccharide (LPS) of bacteria, and further destabilize the cytoplasmic membrane (Wiese et al., 2003; Moffatt et al., 2010). This strong binding is anticipated not to drive resistant mutants against this antibiotic (Clausell et al., 2007; Moffatt et al., 2010). However, there are reports wherein resistance to these peptides has been developed by bacteria through modifications in lipid A, mainly by the addition of 4-amino-4-deoxy-L-arabinose (L-Ara4N) and/or phosphoethanolamine (PEtn) (Raetz et al., 2007; Moffatt et al., 2010). This modification promotes the reduction in the net negative charge on LPS, thereby escalating the resistance against antimicrobial agents. Though the precise mechanism of colistin resistance in AB is not known, there are several reports asserting diverse causes of colistin-resistant strains (colR). For instance, it has been reported that mutations in PmrA/PmrB genes (responsible for the expression of L-Ara4N and PEtn transferases) might instigate colR (Adams et al., 2009; Moffatt et al., 2010). However, another study discloses that mutations within the genes essential for lipid A biosynthesis (either *lpxA*, *lpxC*, or *lpxD*) lead to strains losing the ability to produce LPS (Moffatt et al., 2010), which further triggers colR in AB. In addition, as per other reports, mutations in Gyrase subunit A (*gyr*A), Gyrase subunit B (*gyr*B), *par*C, and D-alanyl-D-alanine carboxypeptidase (DacC) genes are expected to play a major role in antimicrobial resistance (AMR) (Park et al., 2011; Ardebili et al., 2015; Mustikaningtyas et al., 2021).

Having explained this, colistin is still the major choice of antibiotic for treating MDR AB infections. This calls for a solution that can breathe new life into this health challenge. One most reasonable and effective alternative tactic to this issue would be by boosting the effectiveness of colistin as the chances of discovering a new class of antibiotics against MDR AB is grim. This can be done by means of potentiators/adjuvant compounds or combination therapy. Combination therapies can reduce the toxicity that might occur in antibiotic monotherapies by sparing the dosage of both antibiotic and the potentiator (MacNair and Brown, 2020). The potentiators could enhance the activity of existing antibiotics against multiple Gram-negative MDR pathogens, anti-biofilm activity, minimal resistance development, and *in vivo* activity (Klobucar and Brown, 2022). The potentiation mechanism might be either membrane permeabilization, inhibition of bacterial efflux pumps, remodeling of cell walls, or stress response (Melander and Melander, 2017; Blankson et al., 2019; Martin et al., 2019; Klobucar and Brown, 2022). Since the outer membrane (OM) of GNB prevents the entry of antibiotics that are otherwise targeted against them, discovering molecules that can increase the permeability of the OM and sensitize bacteria to ineffective antibiotics might be an appreciated move to treat MDR bacterial infections.

In this study, we describe a potentiator developed by our group (Dey et al., 2022) that specifically targets the OM of the bacterial cell. Based on a report (Mitra et al., 2016), wherein certain synthetic guanidinium derivatives have a bactericidal effect against Gram-negative and Gram-positive bacteria, we have developed a formulation based on a cationic guanidinium derivative with a pyridine moiety (AD-L). This AD-L can form hydrogels, which have high water content and can be widely used for the controlled release of materials (Aswathy et al., 2020). Guanidine-based derivatives (GBDs) are reported to be non-toxic, have broad-spectrum antimicrobial activity, and have the excellent biocidal ability (Aleshina et al., 2001; Lee et al., 2006; Qian et al., 2011b). These GBDs exert their antimicrobial mechanism through electrostatic interactions between the cationic guanidine group and the anionic group on the surface of the microbial cell, thereby imposing a charge imbalance and further triggering the breakdown of the OM and finally oozing of the intracellular components (Qian et al., 2011b; Yim et al., 2013). GBDs are highly biocompatible and thus have been widely used in various health applications such as medical (Buxbaum et al., 2006), cosmetic (Kaehn, 2010), water decontamination (Aviv et al., 2016), textile (Qian et al., 2011a), plastic (Lin et al., 2005; Thomassin et al., 2007), food (Yim et al., 2013), and other industries. Guanidine is also reported as a versatile ligand that can form stable complexes with various metal ions. Exploiting this property, we have used AD-L for *in situ* reduction of Ag(I) to nano-particulate Ag(0) and to stabilize such silver nanoparticles (AgNPs) within the gel matrix of AD-L (Santos et al., 2019). Silver has been used since the 19th century to treat microbial infections. However, after the discovery of antibiotics, its usage was reduced (Klasen, 2000). Nonetheless, due to the rise in biocide-resistant strains, the use of silver to treat infections is gaining importance (Kalishwaralal et al., 2010; Narang et al., 2017). However, since ionic silver (Ag +) is easily inactivated by complexation and precipitation, its use is limited (Atiyeh et al., 2007). Thus, zerovalent silver nanoparticles [Ag (0)] have served as a valuable alternative for ionic silver (Sintubin et al., 2009). Exploiting these advantages, in this study, we have reported highly efficient dual functions of AD-L@Ag(0) nano-formulation (NF). The cationic AD-L reduced silver ions to metallic silver *in situ* and also coordinated with the silver nanoparticle *via* its abundant electron donating atoms thereby acting as both a reducing and a stabilizing agent. The hydrogel could be used for the sustained release of AgNPs to induce bactericidal influence on AB with a prolonged inhibition time. The proof-of-concept and characterization of this AD-L@Ag(0) NF has already been communicated in our previous study (Dey et al., 2022). We investigate the potentiating proficiency of this novel NF against colistin in our current study. To the best of our knowledge, the use of guanidine derived NF of Ag(0) has never been documented for potentiation activity.

## Experimental methods

### Strains and growth medium

The bacterial strain used in the present study was AB (ATCC-BAA-2800). The stock solutions of AB strain were prepared in 10% glycerol stock stored at −80°C. Prior to use, the bacterial cells were revived by inoculating in a 1:100 ratio of inoculum, 2× YT (Yeast Extract Tryptone), and kept overnight (ON) at 37°C at 250 rpm. Furthermore, the bacteria were sub-cultured in 2× YT broth (obtained from HiMedia) in a 1:100 ratio and grown to mid-log phase (OD_600_ of 0.6–0.8) for ON with shaking at 37°C at 250 rpm. The original clinical characteristics of the stock were maintained by preparing fresh cultures from the glycerol stocks for each experiment.

### Synthesis of AD-L@Ag(0) nano-formulation and characterization

AD-L@Ag(0) was synthesized in the same way as in our previous study (Dey et al., 2022). Briefly, 4-pyridine-carboxaldehyde (0.675 ml, 7.17 mmol) dissolved in ethanol was added to a solution of Triamino-guanidinium chloride (0.25 g, 2.38 mmol). This mixture was stirred for 6 h at 85°C and then kept at an ambient temperature to yield a yellow precipitate. Furthermore, the precipitate was washed with ethanol-water (1:1, v/v) and dried under a vacuum. The batch was characterized for its physio-chemical properties by XPS, FT-IR, and TEM, as previously reported (Dey et al., 2022).

### Drug-susceptibility assays

The minimum inhibitory concentration (MIC) of colistin against AB was determined using the broth microdilution assay according to Clinical and Laboratory Standards Institute (CLSI) guidelines (CLSI, 2022). Initially, 96-well assay plates were prepared with serial half dilutions of colistin, and furthermore, a fresh culture of AB (10^3^ CFU/ml) was added to each well. The plates were incubated ON at 37°C at 250 rpm. The last columns of the plates served as “no antibiotic” and “full growth” controls. MIC of AD-L@Ag(0) formulation was determined from our previous study (Dey et al., 2022).

### Two-dimensional synergy testing

**FIGURE 1:**
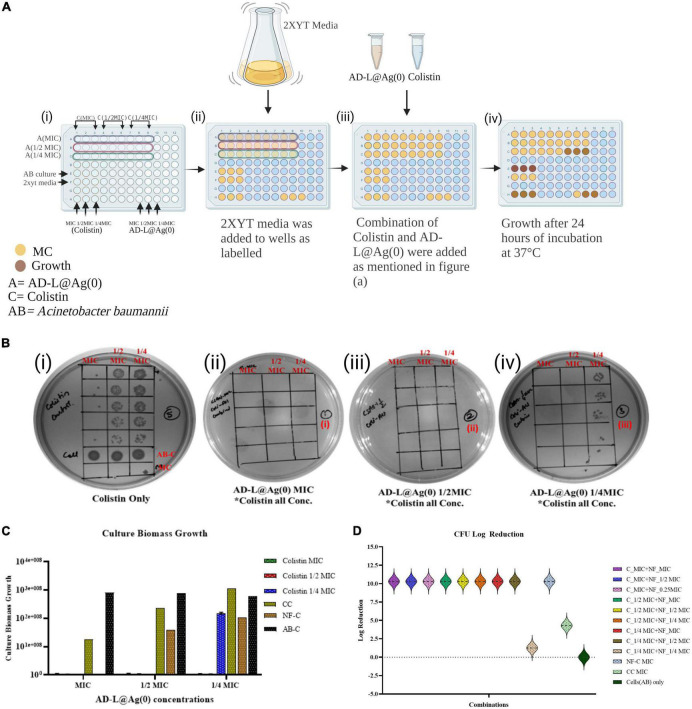
Minimum inhibitory concentration (MIC) calculation: The MIC, ^1/2^ MIC and ^1/4^ MIC of colistin (MIC_col_), of AD-L@Ag(0) nano-formulation [MIC_ADL@Ag(0)_] and that of combination [MIC_Col_ + MIC_ADL@Ag(0)_] against *Acinetobacter baumannii* (AB) was checked using spot assay, CFU count, and by optical density (OD) analysis. The growth of ABin the presence of all the concentrations of colistin confirms resistance. **(A)** Schematic representation of the checkerboard assay at various MICs of colistin and AD-L@Ag(0) to test their synergy or additive effect. **(B)** Spot assays for calculation of MICs: (i) Plate showing growth of ABin the presence of all the MICs of colistin with controls; AB-C, AB control; MC, media control (ii) plate showing effect of the combination of all the MICs of colistin (MIC_col_, ^1/2^ MIC_col_, ^1/4^ MIC_col_) + MIC of AD-L@Ag(0) on the growth of AB (iii) plate showing effect of the combination of all the MICs of colistin (MIC_col_, ^1/2^ MIC_col_, ^1/4^ MIC_col_) + ^1/2^ MIC of AD-L@Ag(0) on the growth of AB (iv) plate showing effect of the combination of all the MICs of colistin (MIC_col_, ^1/2^ MIC_col_, ^1/4^ MIC_col_) + ^1/4^ MIC of AD-L@Ag(0) on the growth of *Acinetobacter baumannii*. **(C)** Culture Biomass Growth measured from absorbance (OD_600_) at various combinations of MICs of colistin and AD-L@Ag(0). The *X*-axis of the histogram represents MIC, ^1/2^ MIC, and ^1/4^ MIC of AD-L@Ag(0), whereas the *Y*-axis shows the culture biomass growth at these MICs. The color bars represent different concentrations of colistin (MIC, ^1/2^ MIC, and ^1/4^ MIC) with controls: CC, colistin control; NF-C, nano-formulation control [AD-L@Ag(0)]; AB-C, *Acinetobacter baumannii* control. **(D)** This figure shows the log reduction in CFU count of AB after treatment with different combinations of colistin and AD-L@Ag(0). The *Y*-axis shows the log reduction of CFU/ml. *X*-axis shows different colors representing various combinations of colistin (MIC, ^1/2^ MIC, and ^1/4^ MIC) with multiple concentrations (MIC, ^1/2^ MIC, and ^1/4^ MIC) of AD-L@Ag(0), along with controls: CC, colistin control; NF-C, nano-formulation control [AD-L@Ag(0)]; AB-C, *Acinetobacter baumannii* control.

The potential synergistic interactions between the colistin and the NF AD-L@Ag(0) were assessed by performing a checkerboard assay (CBA) (Garcia, 2010), which quantifies pairwise drug interactions. For the CBA (Figure 1A), the test NF AD-L@Ag(0) was twofold serially diluted along the rows, while the colistin was twofold serially diluted along the columns to create a matrix in which each well contains a combination of both agents at different concentrations. Different combinations (Table 1) of MIC of colistin and MIC of AD-L@Ag(0) formulation were used to determine whether there is an interaction between colistin and AD-L@Ag(0) formulation or not.

**TABLE 1:** Presence/absence of growth of AB in various combinations of MICs of Colistin + AD-L@Ag(0).

Colistin + AD-L@Ag(0)	Concentration (μ g/ml)	Growth	FICI values
MIC + MIC	2 + 25	No growth	2.0
MIC + ^1/2^ MIC	2 + 12.5	No growth	1.5
MIC + ^1/4^ MIC	2 + 6.25	No growth	1.25
^1/2^ MIC + MIC	1 + 25	No growth	1.5
^1/2^ MIC + ^1/2^ MIC	1 + 12.5	No growth	1.0
^1/2^ MIC + ^1/4^ MIC	1 + 6.25	No growth	0.75
^1/4^ MIC + MIC	0.5 + 25	No growth	1.25
^1/4^ MIC + ^1/2^ MIC	0.5 + 12.5	No growth	0.75
^1/4^ MIC + ^1/4^ MIC	0.5 + 6.25	Growth	0.5

Furthermore, the fractional inhibitory concentration index (FICI) for each AD-L@Ag(0) and colistin combination was measured to analyze the presence or absence of synergism between the two antimicrobial agents. FICI was calculated using the concentrations of the non-turbid wells (indicating low/no growth) in each row and column of the plate, representing the AD-L@Ag(0) and colistin combinations, respectively, at the lowest concentrations that inhibited the growth of AB. This equation used to determine FICI (Berenbaum, 1978) is as follows:

FICI = (MIC_AB_/MIC_A_) + (MIC_BA_/MIC_B_), where,

MIC_AB_: MIC of colistin in combination with AD-L@Ag(0),

MIC_BA_: MIC of AD-L@Ag(0) in combination with colistin together,

MIC_A_: MIC concentrations of colistin alone and,

MIC_B_: MIC concentrations AD-L@Ag(0) alone.

When the FICI mean value was less than or equal to 0.5, it was thought to be synergistic; when it was larger than 0.5 but less than 4, it was thought to be additive or non-interactive; and when it was greater than or equal to 4, it was thought to be antagonistic (Meletiadis et al., 2010).

### Quantification of AB growth

Colony forming unit analysis was done using spot assay (Wang et al., 2017) to quantify bacterial growth (Figure 1). Freshly prepared suspensions of AB containing 1 × 10^3^ CFU/ml were used as an inoculum (100 μl) to be added to wells containing all possible combinations of MICs colistin + AD-L@Ag(0). After 24 h incubation, a spot assay was performed by diluting the wells which showed turbidity (i.e., wells showing growth of bacteria in presence of colistin and AD-L@Ag(0) combination). A small volume (5 μl) of the diluted culture was spotted 5 times on a 2× YT agar plate. The wells with no visible turbidity were not diluted and were directly spotted onto the 2× YT agar plate. All the plates were incubated ON, and the number of colonies, i.e., colony forming units (CFU), were counted directly from spot assay plates using the formula (Whitmire and Merrell, 2012) as follows:


$$\mathrm{CFU}/\mathrm{ml}=\frac{\mathrm{Number}\,\mathrm{of}\,\mathrm{colonies}\times \mathrm{Dilution}\,\mathrm{factor}}{\mathrm{Volume}\,\mathrm{of}\,\mathrm{culture}\,\mathrm{plated}\,\left(\text{mL}\right)}$$

Then, the log reduction of CFU count was calculated by using the formula (Bankier et al., 2018):


$$\mathrm{Log}\,\mathrm{Reduction}:\,\log_{10}\frac{\left(\text{A}\right)}{\left(\text{B}\right)}$$

Or


$$\mathrm{Log}\,\mathrm{reduction}:\log_{10}\left(\text{A}\right)-\log_{10}\left(\text{B}\right)\,$$

where,

A = the number of viable cells in control (AB Only).

B = the number of viable cells after treatment with all the possible combinations of colistin and AL-D@Ag(0), along with NF and CC treatment.

Also, Culture Biomass Growth (CBG) was calculated by determining the optical density (OD_600_) of the cultures. This was done by subtracting the OD of the culture with the OD of the media. Then, the value obtained was multiplied by 2.0 × 10^8^ (Dong-Ju et al., 2012).

## Cell viability assay

### Determination of cellular ATP levels

The effect of the colistin + AD-L@Ag(0) combination on the cellular ATP levels of AB was determined using BacTiter-Glo microbial cell viability assay kit from Promega following the instructions from the manufacturer. Freshly prepared suspensions of AB containing 1 × 10^3^ CFU/ml were added to the 96-well U-bottom plate containing combinations of MICs colistin + AD-L@Ag(0) and kept for 24 h incubation. Next, 25 μl of bacterial suspension from each well was mixed with an equal volume of BacTiter-Glo reagent in a white color 96-well plate and incubated for 5 min at room temperature (RT). Bioluminescence was recorded on Biotek Synergy HT Microplate Reader. Signals represent the mean of duplicates for each measurement. The concentration of cellular ATP was estimated from the ATP standard curve.

### Flow cytometry for estimation of cellular respiration

Overnight cultures of AB were further sub-cultured until we obtained 10^6^ CFU/ml cells. All possible combinations of both drugs were used to treat AB cultures, and these were further used for sample preparation for flow cytometry. One culture treated with colistin only was used as a reference. A culture without treatment with any drug was used as a growth control. Five groups were prepared for Flow cytometry (fluorescence-activated single cell sorting): one was control without any staining, the second was CTC (5-cyano-2,3-ditolyl tetrazolium chloride) stained, the third was DAPI (4′,6-diamidino-2-phenylindole) stained, the fourth one was stained with both dyes, and the fifth was stained with CTC but after fixation with a fixative (4% paraformaldehyde, 2.5% glutaraldehyde, and 0.1 M phosphate buffer having pH 7.2) for 30 min. The fifth group was a negative control for CTC staining. For DAPI staining, cells were first fixed, and for CTC, cells were directly stained.

### Confocal microscopy for viability assessment

Samples prepared for flow cytometry in the fourth group were also used for confocal microscopy. The image was captured in the confocal microscope FV300 Olympus Version 2.4.1.198. CTC and DAPI stained samples were used for confocal imaging. Images were taken at 60X magnification, zoom 4X, and NA 1.35. Channel I DAPI for this PMT voltage is 520 [v], laser transmissivity is 0.8%, and laser ND filter is 10%. Channel II CTC for this PMT Voltage is 490 [v], laser transmissivity is 0.2%, and laser ND filter is 10%. Channel III TD for this PMT Voltage is 350 [v], laser transmissivity is 0.2%, and laser ND filter is 10%. All the images were captured with the same settings.

### Quantitative real-time PCR (qRT-PCR) analysis for mechanistic insight

After incubation at 37°C for 24 h with the treatment of colistin and AD-L@Ag(0) with different MIC concentrations, total RNA was extracted from cells using an RNA extraction Kit (Macherey-Nagel, Germany). After incubation, the culture was homogenized by adding β-mercaptoethanol contacting lysis buffer and mixed by vigorous vortexing. The cell lysate was filtered to remove cell clumps followed by RNA binding to the column. The RNA-bound membrane was further desalted and DNA was digested by rDNase treatment. The membrane was then washed and RNA was eluted in the RNase-free water. All the reagents were provided by the manufacturers in the kit. RNA was quantified by using nanodrop (Thermo, Waltham, MA, United States). cDNA was synthesized by using the iScript cDNA Synthesis kit (Bio-rad, Mumbai), and gene expression was quantified by real-time PCR. RT PCR was performed by using iTaq UniverSYBR Green SMX (Bio-rad, Mumbai) RT-qPCR Kit. Colistin-resistance-related gene expression at the transcript level was checked. GyrA, GyrB, and DacB were utilized in this study. AB-specific 16SrRNA was used as a housekeeping gene control for the expression of normalization.

### Cell cytotoxicity assay

Cytotoxicity of all possible combination MICs of AD-L@Ag(0) formulation + colistin were evaluated against the commonly used epithelial cell line (Vero, E6) using the methyl thiazolyl tetrazolium (MTT) method. The MTT method was performed as per standard protocols by ATCC and as optimized in our previous studies (Dey et al., 2022). MTT assay detects the reduction of yellow tetrazolium (MTT) by metabolically active cells. The resultant purple formazan was measured by using spectrophotometry. Cells were seeded into a 96-well plate in Dulbecco’s Modified Eagle’s medium (D-MEM) with 10% fetal bovine serum (FBS) and 1% penstrep (penicillin 5,000 U/ml + streptomycin 5,000 U/ml). A total of 200 μl of cell suspension at a density of 4 × 10^4^ cells/ml were seeded in the flat bottom 96-well plates with a viability percentage of 97.53. Cells were incubated at 37°C in 5% CO_2_, and the confluency in each well of the 96-well plate was found to be more than 70% after 24 h. Cells were incubated at 37°C in 5% CO_2_ with all possible combinations of AD-L@Ag(0) + colistin. Colistin, AD-L@Ag, one growing control (untreated Vero E6 cells) devoid of antibiotics, and a cytotoxic drug (Docetaxel 44.12 μM) (Altamimi et al., 2021) were kept for controls. Untreated Vero E6 cells were considered as the negative control. After that, 25 μl of MTT reagent (5 mg/ml) was added to each well, and further incubation in a CO_2_ incubator at 37°C for 2 h was done, which resulted in a purple precipitate of Formazan crystals. Formazan crystals were dissolved by using 100 μl DMSO, and then the plates were placed on the shaker at RT for 1 h. Absorbance at 570 nm (reference filter setting was 630 nm) was recorded on the xMark™ Microplate Spectrophotometer (Bio-rad). The experiment was performed in quadruplicate. The cytotoxic effect was checked by comparing growth control, i.e., untreated Vero E6 Cells, with Vero E6 cells treated with different combinations of AD-L@Ag(0) and colistin using analysis by GraphPad Prism version 8.4.3 for Windows, GraphPad Software, San Diego, CA, USA.

### Statistical analysis

All the experiments were performed independently two times with 3–4 replicates in each, and the results are shown as mean with standard error of mean (SEM) as the error bar. Statistical significance (significance level of *p*; 0.05) was calculated by GraphPad Prism software (version 8.0, GraphPad Software, San Diego, CA, USA) using one-way ANOVA, and cytotoxicity data were analyzed using ordinary one-way ANOVA and Dunnett’s multiple Comparisons as a post-test.

## Results

### Characterization of the synthesized formulation

**FIGURE 2:**
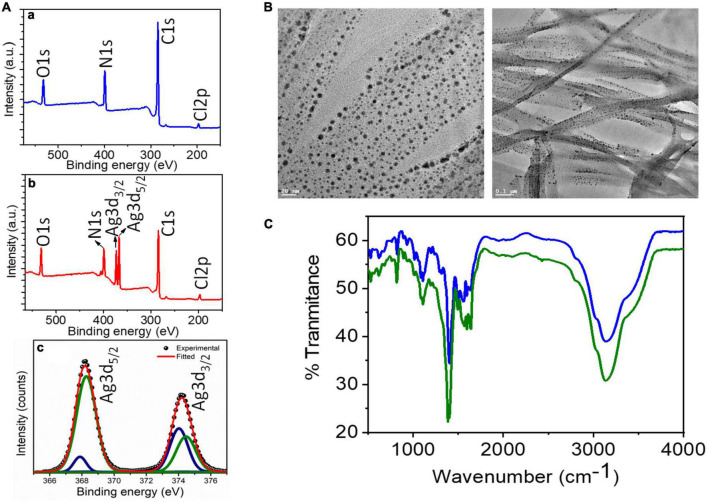
Batch characterization of AD-L@Ag(0): **(A)** XPS analysis: (a,b) XPS survey spectra of AD-L (blue trace), and AD-L@Ag (red trace) respectively. (c) Represents high-resolution deconvoluted spectra collected in the Ag 3d region for AD-L@Ag. **(B)** TEM analysis: TEM images of metallogel from AD-L@Ag(0) showing the gel fibers with *in situ* formed silver nanoparticle at 20 nm and 0.1 μm. **(C)** FT-IR analysis: FT-IR analysis of AD-L@Ag(0) in powder (blue line) and the gel phase (green line).

X-ray photoelectron spectroscopic (XPS) results (Figure 2A) confirm the synthesis of AD-L@Ag(0) by providing the chemical composition and valence state of Ag, C, and N-atoms. The wide energy survey spectrum (wess) of pure ligand AD-L (A) exhibited four peaks which were ascribed to Cl 2p (196.99 eV), C 1 s (284.85 eV), N 1 s (399.13 eV), and O 1 s (532.01 eV) elements, respectively (Figure 2Aa). It is expected that the presence of O 1 s might be due to the presence of a small amount of carbonate counter anion. The wess of silver metal-containing AD-L@Ag(0) gel is shown in Figure 2Ab. The peaks of Cl2p, N 1 s, C 1 s, and O 1 s (present in AD-L) along with Ag 3d confirm the synthesis of AD-L@Ag(0) gel. Furthermore, the deconvoluted spectra of Ag 3d spectrum (Figure 2Ac) are found to split into 3d 5/2 and 3d 3/2 with a spin-orbit splitting of ∼6 eV, which corresponds to unperturbed metallic silver and is typically anticipated for Ag(0) atoms in the nanoparticle core. The XPS results finally confirm the synthesis of the NF. The transmission electron microscopy (TEM) images in Figure 2B shows the synthesized nanoparticles at different magnifications (20 nm and 0.1 μm). The images represent the entangled fibril morphology of the formulation with uniformly distributed Ag nanoparticles on the fibril network. FT-IR analysis that aids in exact structure elucidation and spectra of the compound AD-L@Ag(0) is presented in Figure 2. The presence of the imine linkages (-*C* = N str.) peak at ∼1,380 and 1,595 cm^–1^, indicating a successful reaction between the tri-amino-guanidinium and the 4-pyridinecarboxaldehyde moieties. Additionally, the broad peak at 3,200 cm^–1^ is attributed to the amine functionalities (-N-H str.).

### AB surviving colistin

We first checked the MIC value of colistin, which was 2 μg/ml as reported by CLSI. The MIC can be understood as the lowest concentration of the antibiotic that does not yield any visible growth of the bacteria after 18 h of incubation. However, AB showed persistent growth at this MIC, which is shown in Figure 1Bi. The current scenario of increasing resistance to antibiotics due to mutations indicates the need for substances that can potentiate the activity of the antibiotics. The MIC of AD-L@Ag(0) formulation against AB was calculated to be 25 μg/ml.

### Evaluating AD-L@Ag(0)–colistin treatment combinations on AB growth

We next determined the MIC values for various combinations of colistin with AD-L@Ag(0) formulation and calculated FICI values to establish whether there was evidence of the additive effect of synergy in the combination treatment. The MIC values of colistin with AD-L@Ag(0) combinations were 1.0 and 6.25 μg/ml, respectively (Table 1). These results concluded that only one-fourth of the MIC of the NF in combination with only half MIC of colistin is favorable enough to show a bactericidal effect on AB.

The combination of MICs colistin with AD-L@Ag(0) produced FICI values of 0.5–2 (indicating an additive effect, as described in materials and methods). The results further confirm that at one-fourth MIC of AD-L@Ag(0), the MIC_col_ reduced twofold (reduced to 1 μg/ml, well below the MIC_col_ of 2 μg/ml). These results provide convincing evidence that the AD-L@Ag(0) NF could potentiate the killing effect of colistin for AB at substantially lower concentrations than the individual treatments.

### CFU count for establishing growth inhibition of AB with the combination of colistin and AD-L@Ag(0)

Figures 1Bii, iii show that various combinations of MICs (MIC, ^1/2^ MIC, ^1/4^ MIC) of colistin ranging from 2, 1, and 0.5 μg/ml with the MIC and ^1/2^ MIC of AD-L@Ag(0), i.e., 25 and 12.5 μg/ml, respectively, did not show any growth. Furthermore, it is evident from Figure 1Biv that ^1/4^ MIC (6.25 μg/ml) of AD-L@Ag(0) can potentiate the activity of colistin up to half of its MIC (1 μg/ml). However, it could not show the bactericidal effect when the MIC of colistin was further reduced to one-fourth (0.5 μg/ml). This confirms that AD-L@Ag(0) can potentiate the effect of colistin to which AB became resistant (Figure 1Bi). Out of all combinations, 1.0 μg/ml, i.e., ^1/2^ MIC of colistin, and 6.25 μg/ml, i.e., ^1/4^ MIC of AD-L@Ag(0), were demonstrated as the best potentiation concentrations. Figure 1C shows the histogram view of the calculated CBG confirming the potentiating ability of the developed NF. Similarly, Figure 1D shows the histogram view of the calculated log reduction of CFU confirming the potentiating ability of the developed NF.

### Adenosine triphosphate (ATP) assay for estimation of viability reduction

**FIGURE 3:**
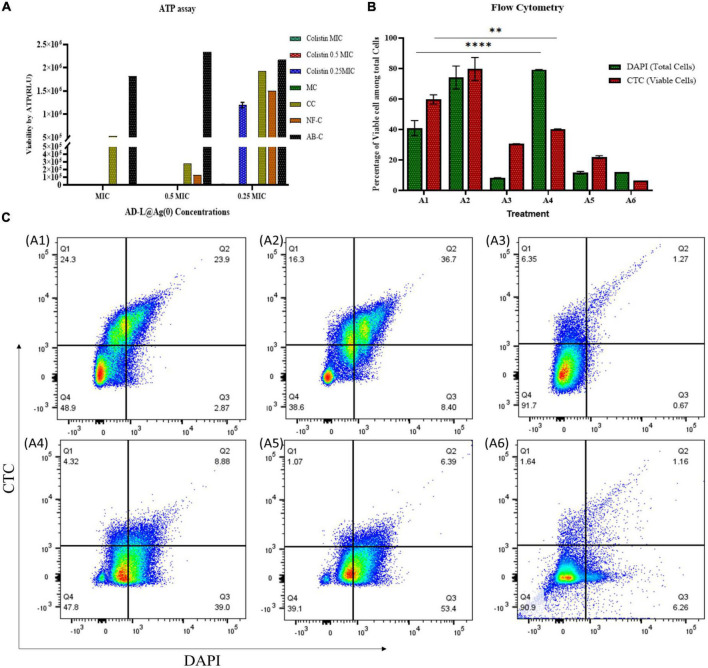
Determination of potentiating ability of AD-L@Ag(0) by estimation of cell viability: The viability of ABin the presence of various combinations of MICs of colistin and AD-L@Ag(0) was analyzed using ATP assay, flow cytometry, and by confocal laser scanning microscopy (CLSM). **(A)** ATP assay: The graph represents presence/absence of ATP production at various combinations of colistin + AD-L@Ag(0). The *x*-axis in the graph represents various concentrations of MICs (MIC, ^1/2^ MIC, ^1/4^ MIC) of AD-L@Ag(0); whereas the *y*-axis represents viability by ATP in terms of RLU (relative light unit). The color bars represent different concentrations of colistin (MIC, ^1/2^ MIC, and ^1/4^ MIC) with controls: CC, colistin control; NF-C, nano-formulation control [AD-L@Ag(0)]; AB-C, *Acinetobacter baumannii* control; MC, media control. The graph suggested similar results as those obtained from CFU and OD calculation. The RLU of ATP at colistin ^1/2^ MIC + AD-L@Ag(0) ^1/4^ MIC was found to be zero indicating bactericidal effect on resistant AB strain. **(B)** Flow cytometry: The viability percentage of ABin various combinations of colistin and AD-L@Ag(0) was analyzed by flow cytometry using DAPI and CTC staining. The *x*-axis represents the treatment of ABat various combinations and the *y*-axis represents the percentage of viable cells among total cells. A1: *Acinetobacter baumannii* control (AB-C), A2: Colistin control (CC), A3: Nano-formulation control [AD-L@Ag(0)] (NF–C), A4: ^1/2^ MIC_col_ + ^1/4^ MIC_AD–L@Ag(0)_, A5: ^1/4^ MIC_col_ + ^1/2^ MIC_AD–L@Ag(0)_, and A6: ^1/4^ MIC_col_ + MIC_AD–L@Ag(0)_. Flow cytometry analysis indicated that at colistin ^1/2^ MIC + AD-L@Ag(0) ^1/4^ MIC (A4), the difference between viable cells (CTC stained) to total cells (DAPI stained) was very huge. This means that the percentage of viable cells was very low when compared to total cells; indicating bactericidal effect at this combination. **(C)** Pseudo-color plot (Flow cytometry): The graph represents four quadrant images with the percentage of cells in each quadrant observed by flow cytometric analysis. Q1: Shows AB cells stained with CTC only, Q2: Shows AB cells stained with both CTC and DAPI, Q3: Shows AB cells stained with DAPI only, Q4: Shows AB cell debris. A1: *Acinetobacter baumannii* control (AB-C), A2: Colistin control (CC), A3: Nano-formulation control [AD-L@Ag(0)] (NF–C), A4: ^1/2^ MIC_col_ + ^1/4^ MIC_AD–L@Ag(0)_, A5: ^1/4^ MIC_col_ + ^1/2^ MIC_AD–L@Ag(0)_, and A6: ^1/4^ MIC_col_ + MIC_AD–L@Ag(0)_. ** (0.0081) Shows the significant difference between AB–C and ^1/2^ MIC_col_ + ^1/4^ MIC_AD–L@Ag(0)_ (TOTAL CELLS). **** (<0.0001) Shows the significant difference between AB–C and ^1/2^ MIC_col_ + ^1/4^ MIC_AD–L@Ag(0)_ (VIABLE CELLS).

Adenosine triphosphate is an energy-transporting molecule that is generally conserved and drives a variety of biological activities essential for the survival of organisms. As a result, ATP is continuously used up since it promotes cell contractile activities, aids membrane transportation, and catalyzes unfavorable reaction dynamics. ATP production mechanisms connected to aerobic respiration and glycolytic activities quickly regenerate ATP to sustain these necessary activities. Since intracellular ATP generation and consumption rates are strictly controlled and consistent during homeostasis, ATP can be used as a substitute for cell viability and vitality (Riss et al., 2016). Hence, to check the viability of bacterial cells in different MIC concentrations, an ATP assay was performed. ATP assay (Figure 3A) shows similar results to those obtained from CFU analysis. The relative fluorescence unit (RLU) of ATP was found to be zero for colistin ^1/2^ MIC + AD-L@Ag(0) ^1/4^ MIC (Figure 3A), which shows that the combined effect of colistin and AD-L@Ag(0) shows a bactericidal effect on resistant AB strain.

### Flow cytometry to assess viability reduction

Flow cytometry was done using CTC and DAPI staining. CTC stain is used for counting and staining actively respiring bacteria. Live bacteria take up CTC and reduce CTC to insoluble formazan (CTC Formazan) (Kobayashi et al., 2012), while dead or inactive bacteria will not be able to do the same. The DAPI has the ability to stain all (live and dead) bacteria by binding to the AT regions in the minor groove of DNA (Tanious et al., 1992). The results (Figure 3B) show that there is a very less number of viable bacteria in the case of colistin ^1/2^ MIC + AD-L@Ag(0) ^1/4^ MIC treated sample compared to the total number of bacteria present in the control. The MIC_col_ shows that the number of viable cells was similar to that of the total cell number present. In the case of MIC_*AD–L@Ag(*0)_, the total cell number as well as the number of viable cells, both were low, and this shows the bactericidal effect of novel NF [AD-L@Ag(0)]. Pseudo-color plot shows the percentage total cell population of AB in all 4 quadrants and viable cell population in Q1 and Q2 quadrants (Figure 3C).

### Microscopic analysis for morphology and viability analysis

**FIGURE 4:**
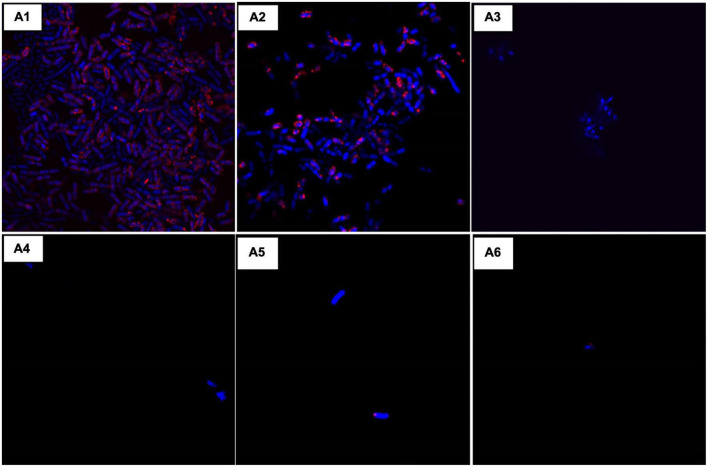
Confocal laser scanning microscopy (CLSM): Morphological changes when using the combination of AD-L@Ag(0) + colistin were observed using CLSM and compared with the controls. Here, **(A1)**: *Acinetobacter baumannii* control (AB-C), **(A2)**: Colistin control (CC), **(A3)**: Nano-formulation control [AD-L@Ag(0)] (NF–C), **(A4)**: ^1/2^ MIC_col_ + ^1/4^ MIC_AD–L@Ag(0)_, **(A5)**: ^1/4^ MIC_col_ + ^1/2^ MIC_AD–L@Ag(0)_, and **(A6)**: ^1/4^ MIC_col_ + MIC_AD–L@Ag(0)_. The results were consistent with our previous observations. Colistin MIC concentration showed visible growth of AB **(A2)** and MIC of AD-L@Ag(0) shows no visible bacteria due to bactericidal effect **(A3)**. Whereas, the combination of two at ^1/2^ MIC_col_ + ^1/4^ MIC_AD–L@Ag(0)_ **(A4)** bacterial growth was inhibited. That is why there is negligible number of bacteria visible under confocal microscope.

Confocal microscopy was done for validating the effect of colistin and AD-L@Ag(0) in different combinations. Consistent with our previous data, in Figure 4, the growing control (A1) shows clear growth of bacteria, while in the case of colistin (A2), the bacterial periphery shows a high amount of CTC, which shows their higher respiring rate to ensure resistance against colistin. At MIC concentration of AD-L@Ag(0) (A3), the absence of bacteria was observed, indicating the bactericidal effect of the NF at MIC. Similarly, in cases A4, A5, and A6, there are no bacteria visible under the microscope after looking at different locations on the same slide, which clearly shows the bactericidal effect of colistin and AD-L@Ag(0) in these combinations. Out of these combinations, A4 (colistin ^1/2^ MIC + AD-L@Ag(0) ^1/4^ MIC) was considered to be a better combination. This is because, at such lower MICs, the additive effect of the potentiator and antibiotic would show low cytotoxicity.

### qPCR analysis

**FIGURE 5:**
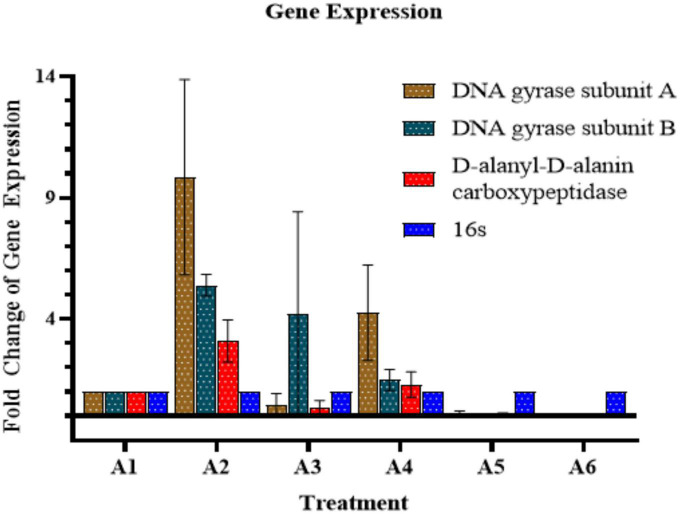
Quantitative Real-time PCR (qRT–PCR) analysis: The graph shows gene expression of GyrA, GyrB, and dacB (presented in *y*-axis) upon different treatments (presented in *x*-axis). Here, A1: *Acinetobacter baumannii* control A–C, A2: Colistin control (CC), A3: Nano-formulation control [AD-L@Ag(0)] (NF–C), A4: ^1/2^ MIC_col_ + ^1/4^ MIC_AD–L@Ag(0)_, A5: ^1/4^ MIC_col_ + ^1/2^ MIC_AD–L@Ag(0)_, and A6: ^1/4^ MIC_col_ + MIC_AD–L@Ag(0)_. A decrease in gene expression when colistin and AD-L@Ag(0) were used in combination was observed in comparison to when only colistin was used.

Quantitative real-time PCR was performed to measure the expression levels of *gyr*A, *gyr*B (introduce negative supercoiling), and DacC (Cell wall biogenesis; peptidoglycan biosynthesis) genes (Table 2). In the case of AB-C (*Acinetobacter baumannii* control, without treatment), the expressions of these genes were found to be at a normal level (A1). While in the case of colistin, we found increased expressions of GyrA, GyrB, and dacB (A2). Furthermore, in the case of AD-L@Ag(0), the expressions of GyrA and dacB were decreased (A3), whereas, in the case of the GyrB gene, the expression was found to be somewhat similar to that of colistin. In the case of ^1/2^ MIC_col_ + ^1/4^ MIC_*AD–L@Ag(*0)_ treated sample (A4), the expressions of these genes show similar patterns as that of colistin, but at comparatively lower levels. This shows that the AD-L@Ag(0) acts on the genes that are involved in the cell wall biogenesis (i.e., dacB) and the ones involved in the negative supercoiling (i.e., GyrA). This concludes that increased expression of these genes is responsible for rendering resistance to colistin in AB. However, in the case of the ^1/2^ MIC_col_ + ^1/4^ MIC_*AD–L@Ag(*0)_ combination, an intermediate expression level of these genes shows that the combination can reduce the resistance efficiency of AB to colistin (Figure 5).

**TABLE 2:** Gene name, function, and antibiotic which target respected genes.

Gene	Gene name	Gene function	Antibiotics
GyrA (Marchese and Debbia, 2016)	Gyrase subunit A	Introduce negative superhelical twists into bacterial chromosomes	Colistin/polymyxin B, levofloxacin, norfloxacin, ofloxacin, and gatifloxacin (DrugBank, 2022)
GyrB (Marchese and Debbia, 2016)	Gyrase subunit B	Introduce negative superhelical twists into bacterial chromosomes	Colistin/polymyxin B and gatifloxacin (DrugBank, 2022)
dacB (Hung et al., 2013)	D-alanyl-D-alanine carboxypeptidase B	Cell shape maintenance	Cefiderocol and meropenem (DrugBank, 2022)

### Cytotoxicity

**FIGURE 6:**
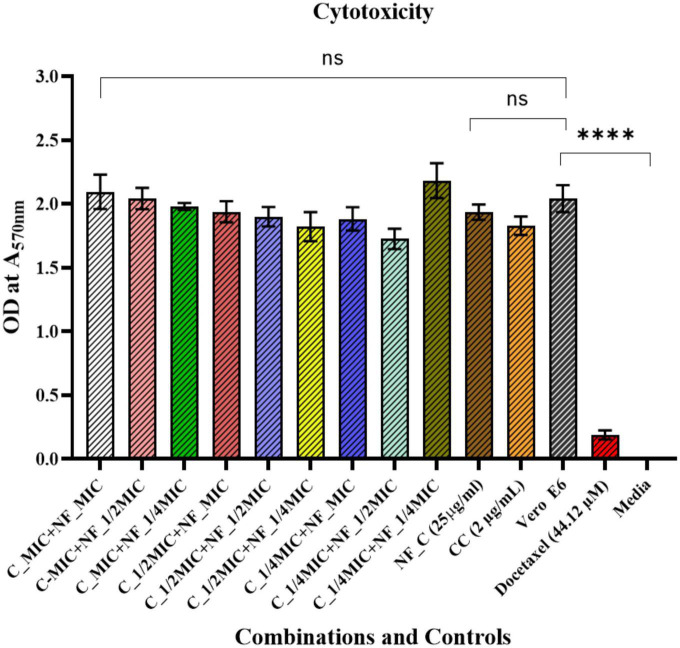
Cytotoxicity assay: A comparison between untreated Vero E6 Cells against treatment with different combinations of AD-L@Ag(0) and colistin is shown here. The *X*-axis represents various combinations of colistin and AD-L@Ag(0), along with colistin control (CC), AD-L@Ag(0) control (NF–C), untreated Vero E6 cells, positive control (Docetaxel),and the *Y*-axis represents the OD values at 570 nm. Results show that there is no significant cytotoxicity found in the case of colistin ^1/2^ MIC and AD-L@Ag(0) ^1/4^ MIC concentration. This is the best combination for potentiation because, at this concentration, it shows less or negligible cytotoxicity to mammalian cells and also demonstrates significant bactericidal effect on colistin-resistant *Acinetobacter baumannii*. **** Shows the significant difference between Live Vero E6 Control and Cytotoxic Drug treated Vero E6 cells (Cytotoxicity Control).

Cytotoxicity assay was performed on mammalian cell lines, i.e., Vero E6 cells. In our previous study, we showed that AD-L@Ag(0) did not have significant toxicity to Vero E6 (Dey et al., 2022). Here, we used different combinations of colistin and AD-L@Ag(0) to assess whether the combination of colistin and AD-L@Ag(0) shows any cytotoxicity toward the mammalian cell line. After performing the MTT assay for cytotoxicity, we found that the colistin ^1/2^ MIC (1.0 μg/ml) + AD-L@Ag(0) ^1/4^ MIC (6.25 μg/ml) combination shows non-significant cytotoxicity to the cells (Figure 6). Hence, this will serve as an ideal combination of colistin and AD-L@Ag(0), which is non-toxic to mammalian cells as well as serve as a good potentiator showing a bactericidal effect on AB.

## Discussion

Antimicrobial resistance is an increasing threat to global healthcare, especially, in the case of the GNB, which has evolved multiple strategies to paralyze antibiotic treatments (Song et al., 2020). Colistin (Polymyxin E), for instance, is considered the “last line of defense” against MDR GNB (Xu et al., 2022). It interacts electrostatically with the OM of GNB and displaces Mg^2+^ and Ca^2+^ ions from their binding sites, thus disrupting the integrity of the membrane and thereby causing cell damage and finally uptake of the antibiotics (Landman et al., 2008; Chawla et al., 2022). Unfortunately, the GNB has developed resistance to this last resort antibiotic colistin. It is reported that in most of the members of the gram-negative *Enterobacteriaceae* family, the colR results from a modification of lipid A component of the LPS (basically plasmid-mediated; MCR; mobile colR) (Dortet et al., 2018), thereby increasing the net charge on LPS. However, in the case of AB, colistin resistance is restricted to chromosome-encoded resistance (Dortet et al., 2018; Partridge et al., 2018). Unfortunately, this colR in AB has emerged due to its increased use (in the case of carbapenem-resistant AB infections), leading to the appearance of pan drug resistance isolates (Brennan-Krohn et al., 2022). Also, the clinical efficacy of colistin is hampered by a low therapeutic index even in susceptible isolates (Brennan-Krohn et al., 2022). Therefore, there is an urgent need for solutions by which the activity of colistin can be restored against resistant AB and also its toxicity can be reduced while retaining the antimicrobial activity. One approach could be the identification of novel molecules, which can increase the permeability of the OM of the GNB, thereby, potentiating the bactericidal activity of colistin against AB. This combination therapy of using novel potentiators along with colistin will not only spare the dose of both the colistin and the potentiator but also will reduce the toxicity associated with colistin (Stokes et al., 2017). There are reports wherein the mechanisms of action of various potentiators have been discussed. An insightful review by Chawla et al. describes the detailed mechanisms by which bacterial resistance can be reversed using various antibiotic potentiators. Some of the strategies include the following: inhibition of conjugative plasmids, plasmid curing, inhibiting efflux pumps, and inhibiting antibiotic resistance enzymes and membrane permeabilizers. Among these approaches, inhibition of conjugating plasmids is responsible for disseminating antimicrobial resistance genes (ARGs) (Chawla et al., 2022). Plasmid curing by agents majorly extracted from plants can remove the ARGs from the bacterial population, thereby reversing plasmid-mediated AMR (Blagden, 1937; Latha et al., 2009; Chandra et al., 2017; Vemuri et al., 2019). The inhibition of the AMR enzymes by using natural inhibitors of enzymes has been used as another alternative approach (Chawla et al., 2022) in combating AMR (Toda et al., 1991; Zhao et al., 2001; Shiota et al., 2004; Schaenzer and Wright, 2020). Another approach for breaking the resistance is by inhibiting efflux pumps, which are mainly responsible for AMR (Chawla et al., 2022). The efflux pumps act by pumping the antibiotics out of the cell, thereby decreasing the concentration of antibiotic inside the cell. A series of natural inhibitors that inhibit the efflux pumps are very much available in literature (Cock, 2012; Chitemerere and Mukanganyama, 2014; He et al., 2019; Mozirandi et al., 2019). Most importantly, compounds acting as membrane permeabilizers in conjunction with antibiotics can play an important role in overcoming resistance. The membrane permeabilizers are generally cationic and amphiphilic in nature targeting OM by interacting with the outer layer ions, lipids, and outer membrane proteins (OMPs). Colistin is one such example of membrane permeabilizer (Burt, 2004; Kwon and Lu, 2006; Landman et al., 2008; Farrag et al., 2019; Klobucar and Brown, 2022). Furthermore, the developed NF is expected to work in the similar way by membrane permeabilization, although the exact mechanism by which AD-L@Ag(0) potentiates the activity of colistin has not been studied. We hypothesize that GBDs exert their antimicrobial mechanism through electrostatic interactions between the cationic guanidine group and the anionic group on the surface of the microbial cell, thereby imposing a charge imbalance and further triggering the breakdown of the OM and finally oozing of the intracellular components (Qian et al., 2011b; Yim et al., 2013). Furthermore, the NF is the combination of guanidinium derivative with pyridine moiety (AD-L) and silver (Ag), where AD-L reduces the silver ion to metallic silver *in situ*. Since it is well-known that nano-silver possesses antimicrobial activity, we proposed that the NF in its hydrogel form releases AgNPs sustainably and induces the bactericidal influence on AB with a prolonged inhibition time.

In the results presented here, we have observed that colistin alone could not show a bactericidal effect against AB at its MIC (2 μg/ml). However, when used in combination with our novel NF [AD-L@Ag(0)], the MIC of colistin is reduced by up to twofold (0.5 μg/ml). This novel potentiator [AD-L@Ag(0)] in combination with colistin produced an FICI value of 0.75 (Table 1), indicating an additive effect (Meletiadis et al., 2010) of both the antimicrobial agents against AB (Figures 1C, D). There are reports in the literature where the additive effects of potentiators with antibiotics have shown tremendous results with drug-resistant isolates (Bezerra dos Santos et al., 2015; Courtney et al., 2017; Puño-Sarmiento et al., 2020).

**FIGURE 7:**
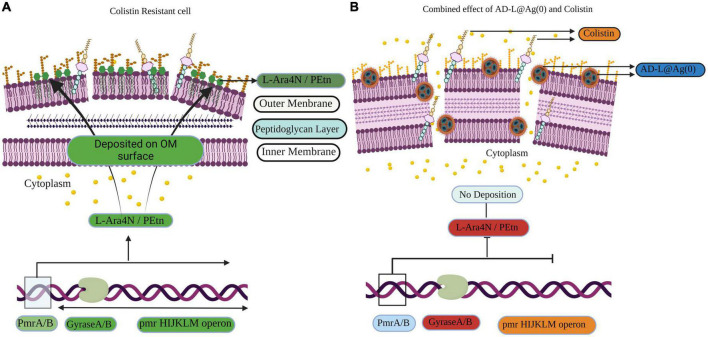
Schematic representation of the induction of colistin resistance as well as reversal of colistin resistance on *Acinetobacter baumanni*. **(A)** Representation of the gene expression in colistin resistant cell: Here green color shows the upregulation of genes, e.g., GyraseA/B and pmrHIJKLM operon. pmrHIJKLM operon codes for phosphoethanolamine (PEtn) or 4-amino-4-deoxy-L-arabinose (L-Ara4N). PEtn gets deposited in the Lipid A layer, and this leads to a decrease in net negative charge, which ultimately leads to colistin resistance. **(B)** Representation of the gene expression in the reversal of colistin resistance when colistin + AD-L@Ag(0) combination is used: Here, it is shown how AD-L@Ag(0) affects GyraseA/B activity, which leads to inhibition of pmrHIJKLM operon. Red color shows the downregulation of genes GyraseA/b genes when the combination was used. GyraseA/B are important for negative supercoiling during processes like DNA replication, transcription, and translation. Hence, its downregulation ultimately hinders the expression of pmrHIJKLM operon. So, there is no PEtn or L-Ara4N production and there is no change in Lipid A composition and net charge present on OM. This leads colistin and AD-L@Ag(0) to exert their bactericidal effect by disrupting the cell membrane, which leads to cell death.

In the case of colistin, the resistance is attributed to the modifications in LPS. This is done by adaptations in Lipid A *via* biosynthesis of PEtn or L-Ara4N. PmrA/B are the transcription factors that control the expression of pmrHIJKLM operon (Figure 7), which ultimately synthesizes PEtn or L-Ara4N on its expression (Falagas et al., 2010; Needham and Trent, 2013; Olaitan et al., 2014). GyraseA/B are the topoisomerases that introduce negative supercoiling in the DNA and are hence important for cellular processes such as replication, transcription, and translation (Reece and Maxwell, 1991). In the case of AD-L@Ag(0) treatment, we observed that the expression of GyraseA decreased to a negligible value, while in the case of colistin, we found a very high expression of GyraseA and B (Figure 5). When the combinations of colistin and AD-L@Ag(0) were analyzed, we found that there is a decrease in the expression of GyraseA and B as compared to the colistin-treated sample. With these observations, it can be hypothesized that during combination treatment, AD-L@Ag(0) affects the GyraseA activity of the cell, which ultimately will not allow the transcription machinery to go forward to transcribe and translate pmrHIJKLM operon and there is no PEtn or L-Ara4N present on the surface of OM of bacteria. This means there are no modifications on Lipid A when the bacterium is treated with colistin and AD-L@Ag(0) combinations (Figure 7). Therefore, colistin works efficiently and disrupts GNB’s cell membrane, due to which cellular component comes out of the cell and ultimately leads to bacterial death.

The Increasing incidence of Colistin Resistance in AB, which ultimately burdens the healthcare service providers. In this study, we have introduced a novel antibiotic adjuvant that can potentiate an antimicrobial agent against resistant bacteria. We posit that AD-L@Ag(0) and analogs thereof represent attractive leads as adjuvants to address the emerging threat of pan-resistant Gram-Negative infections and subsequently the inevitable AMR.

## Conclusion

We showed that guanidinium and silver [AD-L@Ag(0)] based NF, used in combination with colistin, exhibited antibacterial activity against MDR AB, even at a sub-MIC level of AD-L@Ag. Therefore, it acts as an additive potentiator and can enable effective lower dosing of colistin against colistin-resistant AB.

## Data availability statement

The original contributions presented in this study are included in the article/supplementary material, further inquiries can be directed to the corresponding authors.

## Author contributions

SC conceived the study plan, designed the experiments, analyzed the data, and drafted the manuscript. AD and SK contributed to the study plan and manuscript drafting. DK, CS, MY, and PJ performed the experiments, analyzed the data, and contributed to drafting the manuscript. PP and ST performed the experiments and validations. All authors read and approved the final version of the manuscript.

## References

[B1] Adams M. D. Nickel G. C. Bajaksouzian S. Lavender H. Murthy A. R. Jacobs M. R. (2009). Resistance to colistin in *Acinetobacter baumannii* associated with mutations in the PmrAB two-component system. *Antimicrob. Agents Chemother.* 53 3628–3634. 10.1128/AAC.00284-09 19528270 PMC2737849

[B2] Aleshina E. Y. Yudanova T. Skokova I. (2001). Production and properties of polyvinyl alcohol spinning solutions containing protease C and polyhexamethylene guanidine. *Fibre Chem.* 33 421–423. 10.1023/A:1015039328620

[B3] Altamimi A. S. El-Azab A. S. Abdelhamid S. G. Alamri M. A. Bayoumi A. H. Alqahtani S. M. (2021). Synthesis, anticancer screening of some novel trimethoxy quinazolines and VEGFR2, EGFR tyrosine kinase inhibitors assay; molecular docking studies. *Molecules* 26:2992. 10.3390/molecules26102992 34069962 PMC8157871

[B4] Ardebili A. Lari A. R. Beheshti M. Lari E. R. (2015). Association between mutations in gyrA and parC genes of *Acinetobacter baumannii* clinical isolates and ciprofloxacin resistance. *Iran. J. Basic Med. Sci.* 18 623–626. 26221488 PMC4509960

[B5] Aswathy S. Narendrakumar U. Manjubala I. (2020). Commercial hydrogels for biomedical applications. *Heliyon* 6:e03719. 10.1016/j.heliyon.2020.e03719 32280802 PMC7138915

[B6] Atiyeh B. S. Costagliola M. Hayek S. N. Dibo S. A. (2007). Effect of silver on burn wound infection control and healing: Review of the literature. *Burns* 33 139–148. 10.1016/j.burns.2006.06.010 17137719

[B7] Aviv O. Amir N. Laout N. Ratner S. Basu A. Domb A. J. (2016). Poly (hexamethylene guanidine)-poly (ethylene glycol) solid blend for water microbial deactivation. *Polym. Degrad. Stab.* 129 239–245. 10.1016/j.polymdegradstab.2016.04.020

[B8] Bankier C. Cheong Y. Mahalingam S. Edirisinghe M. Ren G. Cloutman-Green E. (2018). A comparison of methods to assess the antimicrobial activity of nanoparticle combinations on bacterial cells. *PLoS One* 13:e0192093. 10.1371/journal.pone.0192093 29390022 PMC5794139

[B9] Berenbaum M. (1978). A method for testing for synergy with any number of agents. *J. Infect. Dis.* 137 122–130. 10.1093/infdis/137.2.122 627734

[B10] Bezerra dos Santos A. Araújo T. Nascimento da Silva L. da Silva C. (2015). Organic extracts from *Indigofera suffruticosa* leaves have antimicrobial and synergic actions with erythromycin against *Staphylococcus aureus*. *Front. Microbiol.* 6:13. 10.3389/fmicb.2015.00013 25699022 PMC4313721

[B11] Blagden C. (1937). A dictionary of the economic products of the malay peninsula. By JH Burkill, with contributions by William Birtwistle, Frederick W. Foxworthy, JB Scrivenor, and JG Watson. 2 vols. 9× 6. pp. xi+ 2402. London: Published on behalf of the governments of the straits settlements and federated malay states by the crown agents for the colonies, 1935. 30s. *J. R. Asiat. Soc.* 69 134–135. 10.1017/S0035869X00096325

[B12] Blankson G. Parhi A. K. Kaul M. Pilch D. S. LaVoie E. J. (2019). Structure-activity relationships of potentiators of the antibiotic activity of clarithromycin against *Escherichia coli*. *Eur. J. Med. Chem.* 178 30–38. 10.1016/j.ejmech.2019.05.075 31173969 PMC6679755

[B13] Brennan-Krohn T. Grote A. Rodriguez S. Kirby J. E. Earl A. M. (2022). Transcriptomics reveals how minocycline-colistin synergy overcomes antibiotic resistance in multidrug-resistant *Klebsiella pneumoniae*. *Antimicrob. Agents Chemother.* 66:e0196921. 10.1128/aac.01969-21 35041511 PMC8923212

[B14] Burt S. (2004). Essential oils: Their antibacterial properties and potential applications in foods—a review. *Int. J. Food Microbiol.* 94 223–253. 10.1016/j.ijfoodmicro.2004.03.022 15246235

[B15] Buxbaum A. Kratzer C. Graninger W. Georgopoulos A. (2006). Antimicrobial and toxicological profile of the new biocide Akacid plus^®^. *J.Antimicrob. Chemother.* 58 193–197. 10.1093/jac/dkl206 16751199

[B16] Centers for Disease Control and Prevention [CDC] (2019). *CDC. Antibiotic resistance threats in the United States.* CDC: Atlanta, GA.

[B17] Chandra H. Bishnoi P. Yadav A. Patni B. Mishra A. P. Nautiyal A. R. (2017). Antimicrobial resistance and the alternative resources with special emphasis on plant-based antimicrobials—a review. *Plants* 6:16. 10.3390/plants6020016 28394295 PMC5489788

[B18] Chawla M. Verma J. Gupta R. Das B. (2022). Antibiotic potentiators against multidrug-resistant bacteria: Discovery, development, and clinical relevance. *Front. Microbiol.* 13:887251. 10.3389/fmicb.2022.887251 35847117 PMC9284026

[B19] Chitemerere T. A. Mukanganyama S. (2014). Evaluation of cell membrane integrity as a potential antimicrobial target for plant products. *BMC Complement. Alternat. Med.* 14:278. 10.1186/1472-6882-14-278 25078023 PMC4124163

[B20] Clausell A. Garcia-Subirats M. Pujol M. Busquets M. A. Rabanal F. Cajal Y. (2007). Gram-negative outer and inner membrane models: Insertion of cyclic cationic lipopeptides. *J. Phys. Chem. B* 111 551–563. 10.1021/jp064757+ 17228913

[B21] CLSI (2022). *Performance standards for antimicrobial susceptibility testing*, 32nd Edn. Wayne, PA: CLSI.

[B22] Cock I. E. (2012). Antimicrobial activity of *Callistemon citrinus* and *Callistemon salignus* methanolic extracts. *Pharmacogn. Commun.* 2 50–57. 10.5530/pc.2012.3.11

[B23] Courtney C. M. Goodman S. M. Nagy T. A. Levy M. Bhusal P. Madinger N. E. (2017). Potentiating antibiotics in drug-resistant clinical isolates via stimuli-activated superoxide generation. *Sci. Adv.* 3:e1701776. 10.1126/sciadv.1701776 28983513 PMC5627983

[B24] Dey A. Yadav M. Kumar D. Dey A. K. Samal S. Tanwar S. (2022). A combination therapy strategy for treating antibiotic resistant biofilm infection using guanidinium derivative and nanoparticulate Ag(0) derived hybrid gel conjugate. *Chem. Sci.* 13 10103–10118. 10.1039/D2SC02980D 36128224 PMC9430544

[B25] Dinc G. Demiraslan H. Elmali F. Ahmed S. S. Metan G. Alp E. (2013). Efficacy of sulbactam and its combination with imipenem, colistin and tigecycline in an experimental model of carbapenem-resistant *Acinetobacter baumannii* sepsis. *Chemotherapy* 59 325–329. 10.1159/000356755 24525528

[B26] Dong-Ju K. Seung-Gun C. Sang-Hyup L. Jae-Woo C. (2012). Relation of microbial biomass to counting units for *Pseudomonas aeruginosa*. *Afr. J. Microbiol. Res.* 6 4620–4622. 10.5897/AJMR10.902

[B27] Dortet L. Potron A. Bonnin R. A. Plesiat P. Naas T. Filloux A. (2018). Rapid detection of colistin resistance in *Acinetobacter baumannii* using MALDI- TOF-based lipidomics on intact bacteria. *Sci. Rep.* 8:16910. 10.1038/s41598-018-35041-y 30442963 PMC6237936

[B28] DrugBank (2022). *Leverage the clinical API: Categories*. DrugBank. Available online at: https://go.drugbank.com/drugs (accessed January 3, 2022).

[B29] Evans M. E. Feola D. J. Rapp R. P. (1999). Polymyxin B sulfate and colistin: Old antibiotics for emerging multiresistant gram-negative bacteria. *Ann. Pharmacother.* 33 960–967. 10.1345/aph.18426 10492501

[B30] Falagas M. E. Rafailidis P. I. Matthaiou D. K. (2010). Resistance to polymyxins: Mechanisms, frequency and treatment options. *Drug Resist. Updat.* 13 132–138. 10.1016/j.drup.2010.05.002 20843473

[B31] Farrag H. A. Abdallah N. Shehata M. M. Awad E. M. (2019). Natural outer membrane permeabilizers boost antibiotic action against irradiated resistant bacteria. *J. Biomed. Sci.* 26:69. 10.1186/s12929-019-0561-6 31500622 PMC6732830

[B32] Garcia L. (2010). Synergism testing: Broth microdilution checkerboard and broth macrodilution methods. *Clin. Microbiol. Proced. Handb.* 1 140–162. 10.1128/9781555817435.ch5.12

[B33] Geisinger E. Mortman N. J. Dai Y. Cokol M. Syal S. Farinha A. (2020). Antibiotic susceptibility signatures identify potential antimicrobial targets in the *Acinetobacter baumannii* cell envelope. *Nat. Commun.* 11:4522. 10.1038/s41467-020-18301-2 32908144 PMC7481262

[B34] He J.-M. Sun S.-C. Sun Z.-L. Chen J.-T. Mu Q. (2019). Isovalerylshikonin, a new resistance-modifying agent from *Arnebia euchroma*, supresses antimicrobial resistance of drug-resistant *Staphylococcus aureus*. *Int. J. Antimicrob. Agents* 53 70–73. 10.1016/j.ijantimicag.2018.08.021 30176356

[B35] Hung W.-C. Jane W.-N. Wong H.-C. (2013). Association of a D-alanyl-D-alanine carboxypeptidase gene with the formation of aberrantly shaped cells during the induction of viable but nonculturable *Vibrio parahaemolyticus*. *Appl. Environ. Microbiol.* 79 7305–7312. 10.1128/AEM.01723-13 24056454 PMC3837741

[B36] Kaehn K. (2010). Polihexanide: A safe and highly effective biocide. *Skin Pharmacol. Physiol*. 23(Suppl. 1), 7–16. 20829657 10.1159/000318237

[B37] Kalishwaralal K. BarathManiKanth S. Pandian S. R. K. Deepak V. Gurunathan S. (2010). Silver nanoparticles impede the biofilm formation by *Pseudomonas aeruginosa* and *Staphylococcus epidermidis*. *Colloids Surf. B Biointerfaces* 79 340–344. 10.1016/j.colsurfb.2010.04.014 20493674

[B38] Kim T. Chong Y. P. Park S. Y. Jeon M.-H. Choo E. J. Chung J.-W. (2014). Risk factors for hospital-acquired pneumonia caused by carbapenem-resistant Gram-negative bacteria in critically ill patients: A multicenter study in Korea. *Diagn. Microbiol. Infect. Dis.* 78 457–461. 10.1016/j.diagmicrobio.2013.08.011 24462178

[B39] Klasen H. (2000). Historical review of the use of silver in the treatment of burns. I. Early uses. *Burns* 26 117–130. 10.1016/S0305-4179(99)00108-4 10716354

[B40] Klobucar K. Brown E. D. (2022). New potentiators of ineffective antibiotics: Targeting the Gram-negative outer membrane to overcome intrinsic resistance. *Curr. Opin. Chem. Biol.* 66:102099. 10.1016/j.cbpa.2021.102099 34808425

[B41] Kobayashi T. Mito T. Watanabe N. Suzuki T. Shiraishi A. Ohashi Y. (2012). Use of 5-cyano-2, 3-ditolyl-tetrazolium chloride staining as an indicator of biocidal activity in a rapid assay for anti-*Acanthamoeba agents*. *J. Clin. Microbiol.* 50 1606–1612. 10.1128/JCM.06461-11 22337974 PMC3347101

[B42] Kwon D. H. Lu C.-D. (2006). Polyamines increase antibiotic susceptibility in *Pseudomonas aeruginosa*. *Antimicrob. Agents Chemother.* 50 1623–1627. 10.1128/AAC.50.5.1623-1627.2006 16641427 PMC1472196

[B43] Landman D. Georgescu C. Martin D. A. Quale J. (2008). Polymyxins revisited. *Clin. Microbiol. Rev.* 21 449–465. 10.1128/CMR.00006-08 18625681 PMC2493081

[B44] Latha C. Shriram V. D. Jahagirdar S. S. Dhakephalkar P. K. Rojatkar S. R. (2009). Antiplasmid activity of 1’-acetoxychavicol acetate from *Alpinia galanga* against multi-drug resistant bacteria. *J. Ethnopharmacol.* 123 522–525. 10.1016/j.jep.2009.03.028 19501283

[B45] Lee S. Jin B. S. Lee J. W. (2006). Thermal degradation kinetics of antimicrobial agent, poly (hexamethylene guanidine) phosphate. *Macromol. Res.* 14 491–498. 10.1007/BF03218714

[B46] Li J. Nation R. L. Turnidge J. D. Milne R. W. Coulthard K. Rayner C. R. (2006). Colistin: The re-emerging antibiotic for multidrug-resistant Gram-negative bacterial infections. *Lancet Infect. Dis.* 6 589–601. 10.1016/S1473-3099(06)70580-1 16931410

[B47] Lin C. H. Lin J. C. Chen C. Y. Cheng C. Y. Lin X. Z. Wu J. J. (2005). Feasibility evaluation of chitosan coatings on polyethylene tubing for biliary stent applications. *J. Appl. Polym. Sci.* 97 893–902. 10.1002/app.21844

[B48] MacNair C. R. Brown E. D. (2020). Outer membrane disruption overcomes intrinsic, acquired, and spontaneous antibiotic resistance. *mBio* 11:e01615-20. 10.1128/mBio.01615-20 32963002 PMC7512548

[B49] Marchese A. Debbia E. A. (2016). The role of gyrA, gyrB, and dnaA functions in bacterial conjugation. *Ann. Microbiol.* 66 223–228. 10.1007/s13213-015-1098-x

[B50] Martin S. E. Melander R. J. Brackett C. M. Scott A. J. Chandler C. E. Nguyen C. M. (2019). Small molecule potentiation of Gram-positive selective antibiotics against *Acinetobacter baumannii*. *ACS Infect. Dis.* 5 1223–1230. 10.1021/acsinfecdis.9b00067 31002491 PMC6682313

[B51] Melander R. J. Melander C. (2017). The challenge of overcoming antibiotic resistance: An adjuvant approach? *ACS Infect. Dis.* 3 559–563. 10.1021/acsinfecdis.7b00071 28548487 PMC5798239

[B52] Meletiadis J. Pournaras S. Roilides E. Walsh T. J. (2010). Defining fractional inhibitory concentration index cutoffs for additive interactions based on self-drug additive combinations, Monte Carlo simulation analysis, and in vitro-in vivo correlation data for antifungal drug combinations against *Aspergillus fumigatus*. *Antimicrob. Agents Chemother.* 54 602–609. 10.1128/AAC.00999-09 19995928 PMC2812160

[B53] Mitra S. Kandambeth S. Biswal B. P. Khayum M. A. Choudhury C. K. Mehta M. (2016). Self-exfoliated guanidinium-based ionic covalent organic nanosheets (iCONs). *J. Am. Chem. Soc.* 138 2823–2828. 10.1021/jacs.5b13533 26866697

[B54] Moffatt J. H. Harper M. Harrison P. Hale J. D. Vinogradov E. Seemann T. (2010). Colistin resistance in *Acinetobacter baumannii* is mediated by complete loss of lipopolysaccharide production. *Antimicrob. Agents Chemother.* 54 4971–4977. 10.1128/AAC.00834-10 20855724 PMC2981238

[B55] Mozirandi W. Tagwireyi D. Mukanganyama S. (2019). Evaluation of antimicrobial activity of chondrillasterol isolated from *Vernonia adoensis* (Asteraceae). *BMC Complement. Altern. Med.* 19:249. 10.1186/s12906-019-2657-7 31492140 PMC6731578

[B56] Munoz-Price L. S. Arheart K. Nordmann P. Boulanger A. E. Cleary T. Alvarez R. (2013). Eighteen years of experience with *Acinetobacter baumannii* in a tertiary care hospital. *Crit. Care Med.* 41 2733–2742. 10.1097/CCM.0b013e318298a541 23982021

[B57] Mustikaningtyas D. Widyarti S. Rifa’i M. Widodo N. (2021). Proposed mechanism of antibacterial activity of glutathione by inhibition of the D-alanyl-D-alanine carboxypeptidase enzyme. *Int. J. Pept. Res. Ther.* 27 843–849. 10.1007/s10989-020-10124-5

[B58] Narang J. Malhotra N. Singhal C. Mathur A. Pn A. K. Pundir C. (2017). Detection of alprazolam with a lab on paper economical device integrated with urchin like Ag@ Pd shell nano-hybrids. *Mater. Sci. Eng. C* 80 728–735. 10.1016/j.msec.2016.11.128 28866222

[B59] Needham B. D. Trent M. S. (2013). Fortifying the barrier: The impact of lipid A remodelling on bacterial pathogenesis. *Nat. Rev. Microbiol.* 11 467–481. 10.1038/nrmicro3047 23748343 PMC6913092

[B60] Olaitan A. O. Morand S. Rolain J.-M. (2014). Mechanisms of polymyxin resistance: Acquired and intrinsic resistance in bacteria. *Front. Microbiol.* 5:643. 10.3389/fmicb.2014.00643 25505462 PMC4244539

[B61] Papathanakos G. Andrianopoulos I. Papathanasiou A. Priavali E. Koulenti D. Koulouras V. (2020). Colistin-resistant *Acinetobacter baumannii* bacteremia: A serious threat for critically ill patients. *Microorganisms* 8:287. 10.3390/microorganisms8020287 32093299 PMC7074815

[B62] Park S. Lee K. M. Yoo Y. S. Yoo J. S. Yoo J. I. Kim H. S. (2011). Alterations of gyrA, gyrB, and parC and activity of efflux pump in fluoroquinolone- resistant *Acinetobacter baumannii*. *Osong Public Health Res. Perspect.* 2 164–170. 10.1016/j.phrp.2011.11.040 24159468 PMC3767088

[B63] Partridge S. R. Di Pilato V. Doi Y. Feldgarden M. Haft D. H. Klimke W. (2018). Proposal for assignment of allele numbers for mobile colistin resistance (mcr) genes. *J. Antimicrob. Chemother.* 73 2625–2630. 10.1093/jac/dky262 30053115 PMC6148208

[B64] Peleg A. Y. Seifert H. Paterson D. L. (2008). *Acinetobacter baumannii*: Emergence of a successful pathogen. *Clin. Microbiol. Rev.* 21 538–582. 10.1128/CMR.00058-07 18625687 PMC2493088

[B65] Puño-Sarmiento J. Anderson E. M. Park A. J. Khursigara C. M. Barnett Foster D. E. (2020). Potentiation of antibiotics by a novel antimicrobial peptide against Shiga toxin producing *E. coli* O157: H7. *Sci. Rep.* 10:10029. 10.1038/s41598-020-66571-z 32572054 PMC7308376

[B66] Qian L. Li X. Sun S. Xiao H. (2011a). Preparation of guanidine polymer and its complex as dual-functional agent for cellulose fibre-based hygiene products. *J. Biobased Mater. Bioenergy* 5 219–224. 10.1166/jbmb.2011.1137

[B67] Qian L. Xiao H. Zhao G. He B. (2011b). Synthesis of modified guanidine-based polymers and their antimicrobial activities revealed by AFM and CLSM. *ACS Appl. Mater. Interfaces* 3 1895–1901. 10.1021/am200094u 21488703

[B68] Qureshi Z. A. Hittle L. E. O’Hara J. A. Rivera J. I. Syed A. Shields R. K. (2015). Colistin-resistant *Acinetobacter baumannii*: Beyond carbapenem resistance. *Clin. Infect. Dis.* 60 1295–1303. 10.1093/cid/civ048 25632010 PMC4462660

[B69] Raetz C. R. Reynolds C. M. Trent M. S. Bishop R. E. (2007). Lipid A modification systems in gram-negative bacteria. *Annu. Rev. Biochem.* 76 295–329. 10.1146/annurev.biochem.76.010307.145803 17362200 PMC2569861

[B70] Reece R. J. Maxwell A. (1991). DNA gyrase: Structure and function. *Crit. Rev. Biochem. Mol. Biol.* 26 335–375. 10.3109/10409239109114072 1657531

[B71] Riss T. L. Moravec R. A. Niles A. L. Duellman S. Benink H. A. Worzella T. J. (2016). “Cell viability assays,” in *Assay guidance manual*, eds Markossian S. Grossman A. Brimacombe K. (Bethesda, MD: Eli Lilly & Company).

[B72] Santos M. R. Ferreira S. M. Mendonça P. V. De Bon F. Serra A. C. Coelho J. F. (2019). Guanidine as inexpensive dual function ligand and reducing agent for ATRP of methacrylates. *Polym. Chem.* 10 4944–4953. 10.1039/C9PY00925F

[B73] Schaenzer A. J. Wright G. D. (2020). Antibiotic resistance by enzymatic modification of antibiotic targets. *Trends Mol. Med.* 26 768–782. 10.1016/j.molmed.2020.05.001 32493628

[B74] Shiota S. Shimizu M. Sugiyama J. I. Morita Y. Mizushima T. Tsuchiya T. (2004). Mechanisms of action of corilagin and tellimagrandin I that remarkably potentiate the activity of β−lactams against methicillin-resistant *Staphylococcus aureus*. *Microbiol. Immunol.* 48 67–73. 10.1111/j.1348-0421.2004.tb03489.x 14734860

[B75] Sintubin L. De Windt W. Dick J. Mast J. Van Der Ha D. Verstraete W. (2009). Lactic acid bacteria as reducing and capping agent for the fast and efficient production of silver nanoparticles. *Appl. Microbiol. Biotechnol.* 84 741–749. 10.1007/s00253-009-2032-6 19488750

[B76] Song M. Liu Y. Huang X. Ding S. Wang Y. Shen J. (2020). A broad- spectrum antibiotic adjuvant reverses multidrug-resistant Gram-negative pathogens. *Nat. Microbiol.* 5 1040–1050. 10.1038/s41564-020-0723-z 32424338

[B77] Stokes J. M. MacNair C. R. Ilyas B. French S. Côté J. B. Bouwman C. Farha M. A. (2017). Pentamidine sensitizes Gram-negative pathogens to antibiotics and overcomes acquired colistin resistance. *Nat. Microbiol.* 2:17028. 10.1038/nmicrobiol.2017.28 PMC5360458 28263303

[B78] Tacconelli E. Carrara E. Savoldi A. Harbarth S. Mendelson M. Monnet D. L. (2018). Discovery, research, and development of new antibiotics: The WHO priority list of antibiotic-resistant bacteria and tuberculosis. *Lancet Infect. Dis.* 18 318–327. 10.1016/S1473-3099(17)30753-3 29276051

[B79] Tanious F. A. Veal J. M. Buczak H. Ratmeyer L. S. Wilson W. D. (1992). DAPI (4’, 6- diamidino-2-phenylindole) binds differently to DNA and RNA: Minor-groove binding at AT sites and intercalation at AU sites. *Biochemistry* 31 3103–3112. 10.1021/bi00127a010 1372825

[B80] Thomassin J.-M. Lenoir S. Riga J. Jérôme R. Detrembleur C. (2007). Grafting of poly [2-(tert-butylamino) ethyl methacrylate] onto polypropylene by reactive blending and antibacterial activity of the copolymer. *Biomacromolecules* 8 1171–1177. 10.1021/bm0611228 17348705

[B81] Toda M. Okubo S. Hara Y. Shimamura T. (1991). Antibacterial and bactericidal activities of tea extracts and catechins against methicillin resistant *Staphylococcus aureus*. *Nihon Saikingaku zasshi* 46 839–845. 10.3412/jsb.46.839 1762174

[B82] Velkov T. Roberts K. D. Nation R. L. Thompson P. E. Li J. (2013). Pharmacology of polymyxins: New insights into an ‘old’ class of antibiotics. *Future Microbiol.* 8 711–724. 10.2217/fmb.13.39 23701329 PMC3852176

[B83] Vemuri P. K. Dronavalli L. Nayakudugari P. Kunta A. Challagulla R. (2019). Phytochemical analysis and biochemical characterization f terminalia chebula extracts for its medicinal use. *Biomed. Pharmacol. J.* 12 1525–1529. 10.13005/bpj/1783

[B84] Wang J. Woo M. Yan C. (2017). Spot plating assay for the determination of survival and plating efficiency of *Escherichia coli* in sub-MIC levels of antibiotics. *JEMI Methods* 1 26–29.

[B85] Whitmire J. M. Merrell D. S. (2012). “Successful culture techniques for *Helicobacter species*: General culture techniques for *Helicobacter pylori*,” in *Helicobacter species* (Totowa, NJ: Humana Press), 17–27. 10.1007/978-1-62703-005-2_4 23015487

[B86] Wiese A. Gutsmann T. Seydel U. (2003). Towards antibacterial strategies: Studies on the mechanisms of interaction between antibacterial peptides and model membranes. *J. Endotoxin Res.* 9 67–84. 10.1179/096805103125001441 12803879

[B87] Xu M. Yao Z. Zhao Y. Shi S. Sun Y. Feng L. (2022). Naringenin restores colistin activation against colistin-resistant gram-negative bacteria in vitro and in vivo. *Front. Microbiol.* 13:916587. 10.3389/fmicb.2022.916587 35992710 PMC9382302

[B88] Yim J. H. Fleischman M. S. Rodriguez-Santiago V. Piehler L. T. Williams A. A. Leadore J. L. (2013). Development of antimicrobial coatings by atmospheric pressure plasma using a guanidine-based precursor. *ACS Appl. Mater. Interfaces* 5 11836–11843. 10.1021/am403503a 24164174

[B89] Zhao W.-H. Hu Z.-Q. Okubo S. Hara Y. Shimamura T. (2001). Mechanism of synergy between epigallocatechin gallate and β-lactams against methicillin-resistant *Staphylococcus aureus*. *Antimicrob. Agents Chemother.* 45 1737–1742. 10.1128/AAC.45.6.1737-1742.2001 11353619 PMC90539

